# The stigma of clean dieting and orthorexia nervosa

**DOI:** 10.1186/s40337-017-0168-9

**Published:** 2017-08-25

**Authors:** Suzanne M. Nevin, Lenny R. Vartanian

**Affiliations:** School of Psychology, UNSW Sydney, Sydney, Australia

**Keywords:** Social stigma, Eating disorders, Orthorexia, Anorexia, Control, Blame

## Abstract

**Background:**

Although the stigma of eating disorders such as anorexia has been well established, little is known about the social consequences of “clean dieting” and orthorexia nervosa. In two studies, we examined the social stigma of clean dieting and orthorexia.

**Method:**

In Study 1, participants read a vignette describing a woman following a “clean” diet, a woman with anorexia, or a control target (minimal information about the individual). In Study 2, participants read a vignette describing a woman with orthorexia, a woman displaying identical orthorexic behaviors but without the orthorexia label, a woman with anorexia, or a control target. Participants then rated the target individual on a range of measures assessing stereotypes, emotions, and behavioral intentions toward the target.

**Results:**

Study 1 found that the clean-dieting target was evaluated more negatively than the control target on some dimensions, but less negatively than the target with anorexia nervosa. Study 2 found that evaluations of the targets with orthorexia nervosa were more negative than evaluations of a control target, but did not differ from evaluations of the target with anorexia nervosa. Perceptions of the target’s control over her behavior were associated with more positive evaluations (Studies 1 and 2), whereas perceptions of blame and responsibility for the condition were associated with more negative evaluations (Study 2).

**Conclusions:**

Overall, these findings highlight the potential negative social consequences of clean dieting and orthorexia nervosa, and point to perceptions of control and blame as potential mechanisms underlying the stigma of these conditions.

## Plain English summary

Clean dieting trends have grown increasingly popular in recent years, and there has also been an increase in interest in orthorexia nervosa by clinicians and researchers. However, little is known about the potential social consequences of these conditions. The fill this gap, we had participants read about a specific individual and then evaluate that person in terms of their attitudes and beliefs about her, as well as they desire for social distance from that person. In our first study, participants evaluated the woman more negatively if they learned that she was following a clean living diet than if she was not following such a diet, and evaluated her even more negatively if they learned that the same eating behaviors occurred in the context of a clinical eating disorder (anorexia nervosa). In our second study, participants evaluated the woman more negatively if they learned that she suffered from orthorexia nervosa than if she did not suffer from that condition, and just as negatively if she was described as suffering from anorexia nervosa. Overall, these findings highlight the potential negative social consequences of clean dieting and orthorexia nervosa.

## Background

A dieting trend that has become increasingly popular in recent years is a pattern of “clean” eating. Clean eating refers to eating behaviors that are centered on proper nutrition, restrictive eating patterns, and strict avoidance of foods considered to be unhealthy or impure [1]. Some examples of such diets include the Low Carb High Fat, Super Healthy Family, the Paleo diet and the Raw Food diet [2]. Although these diet programs claim to confer a range of health benefits, they have garnered substantial criticism from health care professionals due to insufficient empirical validation [1, 3]. In fact, research indicates that these restrictive diets can be harmful to people’s health [4]. For example, the majority of these diets contradict national guidelines for a healthy diet (e.g., [5]) and often involve the omission of certain food groups such as carbohydrates and proteins, which can have adverse medical health ramifications (e.g., iron deficiencies).

Taken to the extreme, adherence to these types of clean diets can result in an obsessional adherence to a clean, pure, and healthy diet, which has been termed “orthorexia nervosa” [6]. Individuals with orthorexia frequently restrict their diet to a limited number of foods that they believe to be “pure” and “clean”, such as raw vegetables. Extreme restrictive eating behaviors can escalate over time, and are often accompanied by intensified “cleanses” (partial fasts) regarded as “purifying” [6]. These patterns of extreme restrictive and clean dieting can result in malnutrition and weight loss, even though the intrinsic desire to lose weight may be absent in orthorexia [7]. Importantly, the pathological obsession with nutritional quality of food and the restrictive control over food intake demonstrated by individuals with orthorexia can cause significant disruption to their functioning. For example, it is often the case that individuals with orthorexia spend excessive amounts of time planning and researching “pure” foods, which can impair their ability to partake in daily social life, leading to a loss of social relationships [1, 3].

Although orthorexia shares some features with anorexia nervosa, there are also some notable differences. In particular, whereas individuals with anorexia have an intense fear of gaining weight and consequently restrict dietary intake in attempt to lose weight, individuals with orthorexia generally alter their eating patterns in order to enhance their health and to feel “pure” [6, 8]. Further, individuals with orthorexia consider their eating styles to be virtuous and wholesome, citing ethical reasons for their eating practices, and they may even proudly discuss their dietary practices because they believe that their eating behaviors symbolize moral superiority [9]. In contrast, individuals with anorexia typically strive to conceal their restricted food intake [4].

### Social dimensions of eating, dieting and their disorders

There is substantial evidence in the literature that people make judgments of others based on their eating behaviors. For example, research on *consumption stereotypes* shows that people ascribe stereotypical attributes to others based on what those others eat [10, 11]. Of particular relevance to the current context, the available research suggests that people have mixed views of individuals who engage in healthy dieting. On the one hand, individuals described as eating low-fat foods are evaluated more positively on measures of attractiveness, conscientiousness, and morality compared to individuals described as consuming high-fat foods [12–14]. On the other hand, individuals described as following a low-fat diet are perceived as high-strung, unhappy, antisocial, and self-centered [12, 15]. These studies indicate that attitudes toward healthy dieting are ambiguous and, most importantly, emphasize that there may be social repercussions for individuals who engage in healthy dieting. Given the increasing popularity of clean dieting, such social repercussions might be particularly salient.

Beyond these consumption stereotypes, there is also evidence that people hold negative attitudes toward individuals with eating disorders [16–20]. Negative attitudes toward anorexia have been documented within the general population [16], among university students [18], and health-care professionals [21]. People also appear to underestimate the severity of eating disorders (i.e., how distressing these disorders are and how difficult it is to recover), which may contribute to the heightened stigmatization of the condition [16, 22, 23]. In addition to this tendency to trivialize the severity of eating disorders, there is also a common belief that the illness is self-inflicted and under the individuals’ control, and both of these beliefs can potentially account for prejudicial attitudes toward individuals afflicted with the conditions [20, 24]. Importantly, stigma associated with eating disorders (and mental health more generally) is associated with negative outcomes for the stigmatized individuals, the most important of which might be a decrease in help-seeking behaviors [25, 26].

To date, only one study has examined social perceptions of orthorexia. Simpson and Mazzeo [27] had participants read vignettes describing an individual with orthorexia nervosa or a DSM-5 eating disorder (anorexia nervosa, bulimia nervosa, or binge eating disorder). That study found that, although there were some group differences (e.g., orthorexia was seen as less distressing and less likely to evoke sympathy than the other disorders), the stigma toward individuals with orthorexia was quite similar to the stigma toward individuals with other eating disorders. For example, evaluations of the orthorexia target did not differ from evaluations of the other targets in terms of personal characteristics (e.g., boring vs. interesting), how much they are to blame for their condition, or how difficult the disorder would be to treat. Thus, there is some preliminary evidence of a stigma toward orthorexia. However, that initial study did not include a no-disorder control group to establish baseline ratings on the measured characteristics, and did not examine any potential mechanisms underlying the stigma. There are also other stigma-relevant variables that could be of interest that were not examined in that initial study (e.g., affective reactions, behavioral intentions). Thus, further research is needed in order to develop a richer understanding the nature and breadth of stigma toward this condition.

### The present research

Clean dieting trends have grown increasingly popular in recent years, and there has also been an increase in interest in orthorexia nervosa by clinicians and researchers. To date, however, little is known about the potential social consequences of these conditions. The aim of the present research was to investigate social perceptions of clean dieting (Study 1) and of orthorexia nervosa (Study 2). Specifically, we examined attitudes, stereotypes, affective reactions, and behavioral intentions toward individuals who are following a clean diet or who have orthorexia nervosa. The findings of the current research are intended to shed light on the potential social stigma of clean living diets and their more extreme form, orthorexia nervosa.

## Study 1

In this first study, participants were presented with information describing the dietary behaviors of a target individual who was either following a clean diet or had an eating disorder (i.e., anorexia nervosa). A control condition was also included in order to provide a baseline for participants’ perceptions of the target individual. Participants then rated the target individual on a range of measures assessing stereotypes, emotions, and behavioral intentions toward the target. Based on the consumption stereotypes literature, we predicted that targets described as following a clean diet would be evaluated less favorably than would the control target, although perhaps not on all dimensions (given the sometimes mixed views of individuals who follow healthy diets [11]). Furthermore, following from research on the stigma of anorexia, we predicted that the target described as having anorexia nervosa would be evaluated more negatively than would the clean-dieting target.

## Method

### Participants

Participants were adult women based in the United States who were recruited through the Amazon Mechanical Turk (MTurk) website. MTurk is an online data collection method that has been shown to produce data that are comparable in quality and reliability to those provided by student and community samples [28, 29]. Individuals who are registered with MTurk have access to a range of tasks that they can complete for small monetary incentives. They select, of their own volition, which tasks they wish to complete. Participants were excluded if they did not provide completed data or if they failed any of the validity checks (e.g., a question directing participants to choose a specific response). Complete and valid data were available from 149 participants. Their mean age was 35.57 years (*SD* = 11.92), and their mean body mass index (BMI; calculated from self-reported height and weight) was 25.71 kg/m^2^ (*SD* = 6.85). With regard to ethnicity, 76.5% of participants were Caucasian, 10.1% were Asian, 8.7% were African American, 3.4% were Hispanic American, and 1.3% were American Indian.

### Materials and procedure

Participants signed up for a study on “social perceptions”. After providing informed consent, participants were randomly allocated to one of three conditions. In each condition, participants were presented with a vignette providing information about a target individual (Sarah; see Appendix). The vignettes provided general information relating to the target’s lifestyle (e.g., university student, part-time waitress). The experimental conditions also provided information about her eating behaviors (e.g., follows what she calls a “pure and clean” diet, avoids foods she considers unhealthy, such as dairy and gluten), and how her diet impacts on her lifestyle (e.g., finds it challenging to balance time between work, university and meal planning). The behaviors were identical in both of the experimental conditions, but were described as being related to either a “clean living diet” or a clinical eating disorder (i.e., anorexia nervosa). This allowed us to determine whether the framing of the behaviors had an impact on evaluations of the target [30]. In order to provide a baseline measure of participants’ evaluation of the target depicted in the vignette, a control condition was also included that contained background information about the target (e.g., university student, part-time waitress, finds it challenging to balance her time between work and university) but no information was provided about the target’s dieting behaviors. After reading the information provided in the vignette, participants were asked to rate the target on the following measures:

#### Attitudes

Participants’ reported their overall attitudes toward the target using a single item: “How favorable is your attitude toward Sarah?” [31, 32]. Ratings were made on a seven-point scale (1 = *Extremely unfavorable*, 7 = *Extremely favorable*).

#### Characteristics

Perceptions of the target’s personality and behavioral attributes were measured using an adapted version of the Characteristics Scale [33]. The original scale comprised 20-bipolar adjective pairs of positive and negative characteristics (e.g., sophisticated-naïve). Five additional items were included based on previous research on the stigma of eating disorders (e.g., vain-modest [16, 19, 22, 34]). Participants were asked to rate the extent to which they believed the target possessed each trait by moving the marker along an 11-point continuum. Responses to some items were reverse-coded so that higher mean scores indicated more positive characteristics. As with the original scale [33], internal consistency for the composite scale was high in the present study (α = .95).

#### Affective reactions

Participants’ emotional reactions to the target were assessed using the Affective Reaction Scale [33]. This scale is comprised of 10 bipolar adjective pairs (e.g., empathetic-angry) and participants were asked to imagine how they would feel if they interacted with the target. Each item was rated on an 11-point continuum and some items were reverse-coded so that higher mean scores indicated more positive emotional responses. In line with previous research [33], internal consistency in the present study was high (α = .94).

#### Opinions

The opinions scale was adapted from previous research examining stigma of eating disorders [17, 30], and was intended to measure stigmatizing attitudes toward the target (e.g., *“*Sarah is an attention seeker*”*) and her behavior (e.g., *“*Sarah’s behaviors are irritating”). Participants were asked to rate their agreement with 17 statements on a five-point scale (1 *= Strongly disagree*, 5 *= Strongly agree).* Responses to some items were reverse-coded so that higher mean scores indicated more positive opinions (α = .95).

#### Control over behavior

Given that previous research has found that perceptions of controllability play an important role in the stigma of eating disorders (e.g., [16, 34]), participants were asked to indicate the extent to which they agreed with the statement “Sarah is in control of herself and her behaviors”. Ratings were made on a 5-point scale (1 = *Strongly disagree*, 5 = *Strongly agree*).

#### Social distance

The Social Distance Scale [35] was used to assess participants’ willingness to engage in social contact with the target (e.g., “How willing would you be to have Sarah as your neighbor?”). Two additional items were included to capture participants’ willingness to interact more intimately with the target (“spending the day with Sarah”; “going for dinner with Sarah” [30]). Each of the nine items was rated on a four-point scale (1 = *Definitely unwilling*, 4 = *Definitely willing*), and responses were averaged with higher scores indicating a greater willingness to interact with the target. Consistent with previous research [33], internal consistency for the scale was high (α = .92).

As an additional measure of behavioral avoidance, participants also completed an online version of the seating distance task [36]. This procedure involved showing participants an image of a round table surrounded by seven seats, one of which was marked as the target’s seat [32]. Participants were asked to imagine that they were attending a meeting with the target person, and specify which seat they would select for themselves. Responses were coded such that higher scores indicated a greater desire for distance from the target (one seat removed from the target to the left or to the right is coded as “1”; two seats removed is coded as “2”; and three seats removed is coded as “3”). Previous research [32] has shown that scores on this seating distance task were correlated with scores on the social distance scale, supporting the validity of the measure.

### Data analysis

Multivariate Analysis of Variance (MANOVA) was conducted in order to assess whether stigmatizing attitudes toward the target individual varied as a function of condition. Follow-up univariate analyses with post-hoc Tukey tests were then conducted on each of the dependent measures. A separate ANOVA was conducted to determine whether perceptions of the target’s control over her behavior varied by condition. Next, correlational analyses were conducted to examine the association between perceptions of control and each of the dependent variables. Finally, mediation analyses (using the PROCESS macro [37]), were carried out to determine whether there were indirect effects of target condition on any of the dependent variables through perceptions of control over behavior. This approach uses bootstrapping, which involves repeatedly sampling from the data set (in this case, 10,000 bootstrap resamples) to create an approximation of the sampling distribution of the indirect effect and to generate confidence intervals for these effects. Controlling for participants’ age and BMI did not affect the results and these variables are therefore not included in any of the analyses described below.

## Results

The multivariate analysis revealed a significant overall effect of condition on the combination of dependent measures, *F*(14, 282) = 6.08, *p* < .001, η^2^_p_ = .23 (Table 1). Follow-up univariate analyses showed that, compared to participants in the control condition, participants in the clean dieting condition had less positive attitudes toward the target, rated the characteristics of the target less positively, and indicated that they would seat themselves farther away from the target. Participants in the anorexia condition evaluated the target more negatively than did participants in the clean living condition on all measures except for overall attitude and seating distance.

**Table 1:** Mean (SD) for each dependent variable in Study 1

Self-Report ratings	Anorexia (*n* = 45)	Clean dieting (*n* = 46)	Control (*n* = 58)
Attitudes	4.00 (1.69)^a^	4.43 (1.66)^a^	5.67 (0.87)^b^
Characteristics	4.77 (1.63)^a^	5.88 (1.23)^b^	6.69 (1.22)^c^
Affective reactions	4.94 (2.14)^a^	5.92 (2.09)^b^	6.50 (1.23)^b^
Opinions	3.03 (0.40)^a^	3.22 (0.18)^b^	3.22 (0.42)^b^
Social distance	2.68 (0.70)^a^	3.00 (0.65)^b^	3.21 (0.42)^b,c^
Seating distance	1.93 (0.84)^a^	1.67 (0.70)^a,b^	1.38 (0.56)^c^
Controllability	3.50 (0.76)^a^	4.20 (0.58)^b^	4.15 (0.63)^b^

For each dependent variable, means within a row with a different superscript denote significant pairwise differences between conditions at *p* < .05

There was also a significant effect of condition on perceptions of how much control the target had over her behavior, *F*(2, 146) = 24.23, *p* < .001, η^2^_p_ = .24. Contrary to prediction, the target with anorexia was perceived as having *less* control over her behavior (*M* = 2.89, *SD* = 1.17) compared to the clean living target (*M* = 4.09, *SD* = 0.69) and the control target (*M* = 3.97, *SD* = 0.84), *p*s < .001. Ratings for the clean living and control targets did not differ from one another, *p* = .78.

Correlational analyses indicated that greater perceptions of control over behavior were associated with more positive evaluations (*r*s ≥ |.34|, *p*s < .001). Finally, tests of indirect effects showed that there were significant indirect effects of condition on each dependent variable through perceptions of control over behavior (see Table 2).

**Table 2:** Indirect effects of condition on the dependent variables in Study 1

Dependent variable	Contrast	Indirect effect	Std. Error	95% CI
Attitudes	Control vs. clean/anorexia	−0.35	0.12	−0.60, −0.13
	Clean vs. anorexia	−0.84	0.20	−1.29, −0.49
Characteristics	Control vs. clean/anorexia	−0.41	0.14	−0.72, −0.17
	Clean vs. anorexia	−1.02	0.24	−1.57, −0.62
Affective reactions	Control vs. clean/anorexia	−0.41	0.15	−0.76, −0.16
	Clean vs. anorexia	−1.03	0.27	−1.64, −0.56
Opinions	Control vs. clean/anorexia	−0.08	0.03	−0.15, −0.03
	Clean vs. anorexia	−0.20	0.05	−0.31, −0.12
Social distance	Control vs. clean/anorexia	−0.12	0.05	−0.23, −0.05
	Clean vs. anorexia	−0.31	0.08	−0.49, −0.18
Seating distance	Control vs. clean/anorexia	0.10	0.04	0.03, 0.20
	Clean vs. anorexia	0.24	0.09	0.09, 0.44

## Discussion

Study 1 showed that the target described as following a clean-living diet was evaluated more negatively than was the control target, but less negatively than the target described as having an eating disorder who engages in the same behaviors. One unexpected finding was that the target with anorexia was perceived as having *less* control over her behavior in comparison to the other two targets. Furthermore, greater perceived control was associated with more positive evaluations of the target, and also accounted for the effect of target type on the dependent variables. Previous research has shown that perceiving eating disorders to be under a person’s control is associated with more negative attitudes toward that person (e.g., [19]). One potential explanation for the discrepancy between the current results and previous studies is that our study focused on control over one’s behavior, whereas previous research has focused on the responsibility or blame associated with the eating disorder itself. This possibility is addressed in Study 2.

## Study 2

Study 2 extended the findings of Study 1 by focusing on more “extreme” clean dieting behaviors in the form of orthorexia nervosa. Participants read a vignette describing the pathological eating behaviors of a target individual with orthorexia nervosa or with anorexia nervosa (or a control target). Because Study 1 found that the labelling significantly impacted evaluations of the target’s behavior, we included two versions of the orthorexia target, one which was labelled as orthorexia and one which described the same behaviors but was unlabeled. Participants then evaluated the target on the same dependent measures as in Study 1. In this study, in addition to assessing perceptions of the target’s control over her behaviors, participants reported their perceptions of the target’s responsibility for her condition. Following from the results of Study 1, we hypothesized that the target with orthorexia would be evaluated less favorably than would the control target. Based on the findings of Simpson and Mazzeo [27], we predicted that there would be no difference in the evaluations of individuals with orthorexia and anorexia. Finally, we predicted that control over behavior would be associated with more positive evaluations, and that perceived responsibility for the condition would be associated with more negative evaluations of the target.

## Method

### Participants

Participants were 196 women recruited from MTurk. Their mean age was 35.43 years (*SD* = 11.18), and their mean BMI was 26.26 (*SD* = 6.91). The majority of the sample identified as Caucasian (80.6%), 10.2% as African American, 4.6% as Hispanic American, 2.6% as Asian, 1.0% as American Indian, and 1.0% identified as “Other”.

### Materials and procedure

The procedure was the same as in Study 1, with the following exceptions:

#### Vignettes

Four vignettes were included in this study (see Appendix). The vignette describing the individual with orthorexia nervosa was developed based on previous research and in consultation with a registered dietitian with expertise in orthorexia nervosa. The vignette describing the individual with anorexia was developed based on the existing literature and in consultation with a clinical psychology PhD candidate with expertise in eating disorders. The vignettes provided an in-depth description of the target’s beliefs, her eating behaviors, and how her diet impacts on her life (e.g., relationship with family and friends has become strained). Efforts were made to equate the two vignettes in terms of level of clinical impairment. Prior to reading the vignettes, participants in these two conditions were provided with brief information describing the diagnostic features of the relevant disorder. A third experimental condition was included in which participants read the same details as in the orthorexia condition, but were not presented with the diagnostic information and were not told that the behaviors were associated with orthorexia nervosa. Finally, as in Study 1, the control condition included minimal details about the target, and no information was provided about the target’s dieting behaviors.

#### Blame and responsibility for condition

Participants in the orthorexia and anorexia experimental conditions were asked to indicate the extent to which they believed that the target was to blame for her condition: “To what extent do you believe Sarah is to blame for her condition?”, “To what extent do you believe Sarah’s condition is under her personal control?”, and “To what extent do you believe Sarah is responsible for her condition?”. (Blame and responsibility were assessed only for the labelled orthorexia and the anorexia conditions because those were the only groups for which a “condition” was specifically identified.) Each item was rated on an 11-point continuum, with higher mean scores indicated greater levels of blame for the condition (α = .89).

## Results

The multivariate analysis indicated a significant overall effect of experimental condition on the combination of dependent measures, *F*(8, 567) = 7.67, *p* < .001, η^2^_p_ = .20 (Table 3). Follow-up univariate analyses showed that the three disordered eating groups were evaluated less favorably than was the control target on all measures (except the seating distance task); there were no differences in evaluations among any of the disordered eating groups.

**Table 3:** Mean (SD) for each of the dependent variables in Study 2

Dependent variable	Orthorexia (*n* = 51)	Anorexia (*n* = 49)	Unlabeled (*n* = 47)	Control (*n* = 49)
Attitudes	4.14 (1.47)^a^	4.18 (1.20)^a^	4.11 (1.45)^a^	5.49 (0.89)^b^
Characteristics	4.44 (1.12)^a^	4.42 (0.92)^a^	4.62 (1.21)^a^	6.70 (1.06)^b^
Affective reaction	4.34 (1.70)^a^	4.08 (1.46)^a^	4.34 (1.64)^a^	6.44 (1.44)^b^
Opinions	2.73 (0.52)^a^	2.68 (0.48)^a^	2.84 (0.59)^a^	3.76 (0.46)^b^
Social distance	2.69 (0.76)^a^	2.86 (0.65)^a^	2.79 (0.74)^a^	3.21 (0.39)^b^
Seating distance	1.65 (0.72)^a^	1.53 (0.58)^a^	1.74 (0.74)^a^	1.47 (0.68)^a^

For each dependent variable, means within a row with a different superscript are significant different at *p* < .05

### Control over behaviors

Consistent with Study 1, perceptions of the target’s control over her behavior varied by condition, *F*(3, 192) = 21.80, *p* < .001, η^2^_p_ = .25. Post-hoc Tukey analyses indicated that the control target was rated as having significantly more control over her behavior (*M* = 3.78, *SD* = 0.87) than were targets in the experimental conditions (orthorexia: *M* = 2.31, *SD* = 1.26; unlabeled orthorexia: *M* = 2.40, *SD* = 1.25; anorexia: *M* = 2.16, *SD* = 1.07; all *ps* < .001). There were no significant differences among any of the experimental conditions (*p*s > .79).

### Blame and responsibility for condition

A one-way ANOVA revealed that there was no significant difference in the level of blame attributed to the target with orthorexia (*M* = 6.00, *SD* = 2.30) compared to the target with anorexia (*M* = 5.29, *SD* = 2.71), *F*(1, 98) = 1.97, *p* = .16, η^2^_p_ = .02.

### Correlations

Consistent with Study 1, perceived control over behavior was positively correlated with all dependent measures (*r*s > .20, *p*s < .005), except for the seating distance task (*r* = −.04, *p* = .59). In contrast to perceived control over behavior, blame for the target’s condition was *negatively* correlated with all of the dependent measures (*r*s > −.25, *p*s ≤ .01), except for the seating distance task (*r* = .18, *p* = .07).

### Tests of indirect effects

The test of mediation for perceptions of control over behavior specifically contrasted the control condition with the three experimental conditions. There were significant indirect effects only for characteristics and opinions (see Table 4). Because perceptions of blame did not differ between the orthorexia and anorexia conditions, no tests of indirect effects were conducted.

**Table 4:** Indirect effects of condition on the dependent variables in Study 2

Dependent variable	Indirect effect	Std. Error	95% CI
Attitudes	−0.05	0.13	−0.31, 0.22
Characteristics	−0.38	0.11	−0.63, −0.18
Affective reactions	−0.29	0.16	−0.63, 0.01
Opinions	−0.23	0.06	−0.36, −0.13
Social distance	−0.08	0.06	−0.20, 0.05
Seating distance	−0.01	0.07	−0.02, 0.03

## Discussion

Study 2 extended the findings of Study 1 by showing that the target with orthorexia was consistently evaluated less favorably than was the control target, and this was true regardless of whether the label “orthorexia” was applied to the target’s behavior of not. Interestingly, evaluations of the targets with orthorexia did not differ from the target with anorexia on any of the outcome measures. Follow-up correlational analyses indicated that greater perceived control over behavior was associated with more positive evaluations, but that greater levels of blame for the eating disorder were associated with more negative evaluations of the target individual.

## General discussion

The primary aim of the present research was to investigate social perceptions of “clean” dieting and orthorexia nervosa. Study 1 found that participants evaluated the clean dieting target less favorably on certain dimensions, suggesting that there may be social costs to clean dieting. This finding extends previous research on consumption stereotypes indicating that individuals who consume healthy diets are seen as less socially appealing [11]. Interestingly, these findings seem to diverge from other research showing that obese individuals (and even non-obese individuals) are evaluated more favorably when they engage in efforts to eat a healthy diet and exercise regularly [38, 39]. It may be that the degree of obsessionality and sense of moral superiority that accompanies clean-living diets results in more negative evaluations than does engaging in a “normal” healthy lifestyle.

Study 2 extended the findings of Study 1 by showing that the discrepancy between the control target and the target with orthorexia was even more pronounced than it was for the target who was engaged in the clean-living diet. Importantly, in Study 2, the targets with orthorexia (labelled), with unlabeled orthorexia, and with anorexia were all evaluated equally negatively. Although the present study may not have been sufficiently powered to detect small differences between the anorexia and orthorexia conditions, it can nonetheless be argued that evaluations of these conditions are more similar than they are different. These findings add to prior literature showing that anorexia is evaluated negatively (e.g., [16, 20, 22]), and suggest that these negative evaluations can extend to other types of eating disorders. Our findings are also consistent with those of Simpson and Mazzeo [27] who found that orthorexia was associated with a degree of stigma that is similar to the stigma of anorexia and bulimia.

The current studies also examined whether perceived control and responsibility could explain judgments of the target individuals. In both studies, the targets with eating disorders were perceived as having less control over their behavior than was the control target. It is noteworthy, then, that despite the fact that participants recognized that the behavior of individuals with an eating disorder might not be completely under their control, participants still expressed stigmatizing attitudes toward those individuals. Indeed, both studies found that less perceived control was associated with more negative evaluations. Although these findings appear to be in contrast to earlier work on attributions and stigma [19], the focus on control over one’s behaviors instead of responsibility for one’s condition can potentially explain the discrepancy. Indeed, Study 2 found that the degree of blame that participants attributed to anorexia and orthorexia was associated with more negative evaluations of those individuals. Together, these findings highlight a distinction between control over one’s behavior and blame for a condition that should be considered in future research on attributions and stigma of eating disorders.

Our findings may have some practical implications. For example, it could be important for clinicians to recognize the potential social consequences of clean living and orthorexia because the negative attitudes of others could exacerbate social impairments endured by individuals with disordered eating. Psychoeducation around the stigma of extreme restrictive eating might also assist the client in motivating change. Of course, it is also possible that drawing attention to the negative perceptions of others might increase defensiveness, shame, and internalized stigma. Thus, care needs to be taken in focusing on the stigmatizing aspects of these conditions. Also of potential practical relevance is the finding from previous research that individuals with extreme dieting behaviors often adopt a self-righteous attitude regarding to their food intake and consider their eating behaviors to be morally superior to other individuals’ dietary practices [4, 40]. It is therefore conceivable that individuals suffering from these conditions may be particularly resistant to treatment. Thus, unpacking the social stigma associated with these pathological dieting behaviors could provide motivation to change for individuals with these conditions.

There are some limitations of the present research that should be noted. First, participants evaluated a hypothetical stranger based on limited information. It is unknown whether people would respond in a similar manner in a face-to-face interaction context with someone who is personally known to them (e.g., friend or family member). Thus, future research could use more ecologically-valid approaches to gauging the social consequences clean dieting and orthorexia (as well as other eating disorders). Another limitation is that the targets in the present study were all women, and thus it is unknown whether the same effects would emerged if the targets were men (although some research suggests that there might be similar levels of stigma toward men and women with eating disorders [41]). Finally, our samples included only women and were homogeneous in terms of ethnicity. Thus, it is unknown whether these results would generalize to other populations and demographics (although there is some suggestion that male participants are more stigmatizing of eating disorders than are female participants [27, 42, 23]). These limitations should be addressed in future research.

## Conclusion

The present research provided support for the suggestion that there may be adverse social ramifications for clean dieting behaviors, and found that this effect was particularly pronounced when the behaviors were described in a more extreme manner (i.e., orthorexia nervosa). The present studies also provided preliminary insight into the mechanisms underlying negative evaluations of clean dieting and orthorexia by demonstrating that perceived controllability of behavior predicted more favorable evaluations, whereas perceived blame and responsibility for the condition predicted less favorable evaluations. Developing a better understanding of the stigma toward various forms of disordered eating is an important step toward alleviating the social burden endured by individuals with those conditions.

## Appendix

### Study 1 vignettes:

#### Clean living

Sarah is second year student at University. Her hobbies include shopping and swimming. She has recently become very interested in food and clean living. She follows what she describes as a ‘pure and clean’ diet. She strictly avoids foods that she considers unhealthy, such as dairy and gluten. Sarah has a part time job as a waitress but finds it challenging to balance her time between work, university and meal planning. When Sarah’s friends went to dinner last week she decided not to go because the menu was not suitable to her dietary regime, as the restaurant did not serve ‘clean’ foods. Sarah’s friends think her lifestyle may be dominating her life. Sarah is tired of people questioning her diet and lifestyle. She has to remind herself that her friends are more than likely jealous of her healthy lifestyle and self-discipline.

#### Anorexia nervosa

Sarah is second year student at University. Her hobbies include shopping and swimming. Last year she was diagnosed with anorexia. She follows what she describes as a ‘pure and clean’ diet. She strictly avoids foods that she considers unhealthy, such as dairy and gluten. Sarah has a part time job as a waitress but finds it challenging to balance her time between work, university and meal planning. When Sarah’s friends went to dinner last week, she decided not to go because the menu was not suitable to her dietary regime, as the restaurant did not serve ‘clean’ foods. Sarah’s friends think her lifestyle may be dominating her life. Sarah is tired of people questioning her diet and lifestyle. She has to remind herself that her friends are more than likely jealous of her healthy lifestyle and self-discipline.

#### Control

Sarah is a second year student at University. Her hobbies include shopping and swimming. Sarah has a part-time job as a waitress but finds it challenging to balance her time between work and university.

### Study 2 vignettes

#### Orthorexia nervosa - labelled

*First page:*

Orthorexia nervosa is a medical condition characterized by extreme healthy dieting. Individuals who fit the diagnostic criteria for orthorexia demonstrate a pathological obsession with restrictive dietary rules. Often their obsession with pure and healthy foods escalates over time leading to the elimination of entire food groups, such as carbohydrates or proteins. These behaviors result in physical weight loss, severe malnutrition and a range of medical complications. Individuals with orthorexia experience personal distress and disgust when self-imposed dietary rules are broken. In addition, the condition can result in impairment of social functioning because those suffering from orthorexia become withdrawn and spend excessive amounts of time researching and preparing pure foods.

*Second page:*

Sarah is a 20-year-old third-year university student. Although she was always thin as an adolescent, when she started university, she began to diet in attempt to improve her overall health. She was mostly eliminating fats from her diet, because she read in a nutrition magazine that fats were bad for the metabolism. Sarah gradually began to feel more in control of her health and became more preoccupied with the nutritional quality of foods. Over time her dieting became progressively more restricted and she began to eliminate all foods that contained dairy, grain and animal products. Due to her obsession with healthy eating she now limits what she eats to foods she considers entirely “pure” including fruit and raw vegetables.

Although she is not trying to lose weight, due to her extreme efforts to maintain a healhty diet, Sarah has lost weight, and her periods have now stopped. Despite her increasingly thin and gaunt appearance, Sarah denies that she is unhealthily underweight. She also believes that “impure” foods like bread contain dangerous toxins and artificial chemicals and should be avoided. She spends a lot of time planning and preparing what she will eat. Choosing the “correct” foods can cause her distress and she feels guilty and disgusted when she thinks she has eaten something that she deems “impure”. Her obsession with healthy foods has progressively increased her levels of anxiety and her grades at university have started to slip. She finds it challenging balancing her time between university, taking yoga classes and meal planning.

Last week her friends invited her to dinner but she made an excuse that she couldn’t go because she did not want to have to eat in front of her friends as she thought it would draw attention to her restricted diet. Sarah’s relationship with her family and friends has become strained and they are concerned that her obsession maintaining a healthy diet is dominating her life. However, Sarah maintains a sense of control when she restricts her food intake and feels disgusted by her friends’ eating habits.

#### Anorexia nervosa

*First page:*

Anorexia nervosa is a medical condition characterized by extreme dieting. Individuals who fit the diagnostic criteria for anorexia demonstrate a pathological obsession with restricting their food intake. Often their obsession with dieting and losing weight escalates over time leading to the elimination of entire food groups, such as carbohydrates or proteins. These behaviors result in physical weight loss, severe malnutrition and medical complications. Individuals with anorexia experience personal distress due to an intense fear of gaining weight and a disturbed body image. In addition, the condition can result in impairment of social functioning because those suffering from anorexia become withdrawn and avoid any social gathering that involves food.

*Second page:*

Sarah is a 20-year-old third-year university student. Although she was always thin as an adolescent, when she started university, she began to diet in attempt to improve her overall health. She was mostly eliminating fats from her diet because she read in a nutrition magazine that fats were bad for the metabolism. Through these efforts, Sarah lost weight and girls at university began to compliment her on her appearance. Over time her dieting became progressively more restricted and she began to eliminate all foods that contained dairy, grains and animal products. She now has an intense fear of gaining weight and limits what she eats to foods that she knows are low in calories such as fruit and raw vegetables.

As result of her fixation with losing weight, Sarah’s periods have now stopped and despite her increasingly thin and gaunt appearance, she denies that she is underweight. Due to her distorted body image, she believes she needs to continue dieting to prevent her from gaining weight. Given her obsession with restricting her food intake, she ends up spending a lot of time thinking and planning how she will pretend to eat meals. Mealtimes can result in interpersonal conflict because she often refuses to eat and feels anxious and disgusted when she thinks she has eaten something that will make her gain weight. Her obsession with food and weight has progressively increased her levels of anxiety and her grades at university have started to slip. She finds it challenging balancing her time between university, taking yoga classes and meal planning.

Last week her friends invited her to dinner but she made an excuse that she couldn’t go because she did not want to have to eat in front of her friends as she thought it would draw attention to her restricted diet. Sarah’s relationship with her family and friends has become strained and they are concerned that her obsession with losing weight is dominating her life. However, Sarah maintains a sense of control when she restricts her food intake and feels disgusted by her friends’ eating habits.

#### Control

Sarah is a second year student at university. Her hobbies include shopping and swimming. Sarah has a part-time job as a yoga teacher but finds it challenging to balance her time between work and university.
